# Adapting Coordinated Specialty Care in the Post-COVID-19 Era: Study Protocol for an Integrative Mixed-methods Study

**DOI:** 10.21203/rs.3.rs-452200/v1

**Published:** 2021-04-26

**Authors:** Sapana Patel, Iruma Bello, Leopoldo J. Cabassa, Ilana R. Nossel, Melanie M. Wall, Elaina Montague, Reanne Rahim, Chacku M. Mathai, Lisa B. Dixon

**Affiliations:** Columbia University and the New York State Psychiatric Institute; Columbia University; Brown School Social Work; Columbia Presbyterian Medical Center: Columbia University Irving Medical Center; Columbia University Mailman School of Public Health; New York State Psychiatric Institute; New York State Psychiatric Institute; New York State Psychiatric Institute; Columbia Presbyterian Medical Center: Columbia University Irving Medical Center

**Keywords:** Coordinated Specialty Care, Learning Healthcare System, First Episode Psychosis, Early Psychosis, COVID-19, Telehealth, Youth and Young Adults, Specialized Treatment, Fidelity, Framework for Reporting Adaptations and Modifications-Enhanced

## Abstract

**Background:**

Coordinated Specialty Care (CSC) programs provide evidence-based services for young people with a recent onset of a psychotic disorder. OnTrackNY is a nationally recognized model of CSC treatment in New York state. In 2019, OnTrackNY was awarded a hub within the Early Psychosis Intervention Network (EPINET) to advance its learning health care system (LHS). The OnTrackNY network is comprised of 23 CSC teams across New York state. OnTrack Central, an intermediary organization, provides training and implementation support to OnTrackNY teams. OnTrack Central coordinates a centralized data collection protocol for quality improvement and evaluation of program fidelity and a mechanism to support practice based-research. OnTrackNY sites’ breadth coupled with OnTrack Central oversight provides an opportunity to examine the impacts of the COVID-19 crisis in New York State.

**Methods:**

This project will examine the implications of modifications to service delivery within the OnTrackNY LHS during and after the COVID-19 crisis. We will use the Framework for Reporting Adaptations and Modifications-Enhanced (FRAME) to classify systematically, code, and analyze modifications to CSC services and ascertain their impact. We will utilize integrative mixed methods. Qualitative interviews with multi-level stakeholders (program participants, families, providers, team leaders, agency leaders, trainers (OnTrack Central), and decision-makers at the state and local levels) will be used to understand the process making decisions, information about modifications to CSC services, and their impact. Analysis of OnTrackNY program data will facilitate examining trends in team staffing and functioning, and participant service utilization and outcomes. Study findings will be summarized in a *CSC Model Adaptation Guide*, which will identify modifications as fidelity consistent or not, and their impact on service utilization and care outcomes.

**Discussion:**

A *CSC Model Adaptation Guide* will inform CSC programs, and the state and local mental health authorities to which they are accountable, regarding modifications to CSC services and the impact of these changes on care process, and participant service utilization and outcomes. The guide will also inform the development of tailored technical assistance that CSC programs may need within OnTrackNY, the EPINET network, and CSC programs nationally.

## Background

Coordinated Specialty Care (CSC) programs provide evidence-based services for young people with recent onset of a psychotic disorder. (1) New York State’s program, OnTrackNY, is a nationally recognized model of CSC treatment. (2) The program is implemented by a multidisciplinary team with specialized training, which provides coordinated, evidence-based treatments based on the interests, needs, and preferences of each participant. The program emphasizes assertive outreach and engagement and is offered for an average of two years. OnTrackNY teams offer participants a range of evidence-based treatment options, including individual therapy based on Cognitive Behavioral Therapy for psychosis (CBTp), psychiatric medications, family education and support, peer support, supported education and employment, case management and community support, and a focus on physical wellness and primary care coordination. Teams focus on helping participants attain individual goals typically related to school, work, and relationships. (3) OnTrackNY has demonstrated improvements in symptoms, functioning, hospitalization, and work/school participation. (4) The rapid rise of COVID-19 has created shocks to the health care system, producing numerous rapid changes in behavioral health service delivery, in the absence of guidance from evidence or experience. It is unclear how these changes will impact the ongoing delivery of CSC services and participant outcomes.

OnTrack Central is an intermediary organization (5, 6) responsible for training and implementation support of OnTrackNY programs. It has created systems for multi-level stakeholder engagement, a centralized data collection protocol for quality improvement and evaluation of program fidelity, and a mechanism to support practice based-research. Initial training provided to OnTrackNY teams includes a multi-day in-person introductory training in the principles and practices of the CSC model, followed by remote consultation, and training in data collection forms, procedures, and the data collection platform. Regular, on-going training is also provided for team members, based on their specialty, through frequent individual, collaborative, role and team-based phone calls, monthly care consultation calls, webinars, and use of data and fidelity reports to highlight strengths and identify areas for improvement. (7)

OnTrack Central’s engagement of the OnTrackNY network has revealed how changes due to COVID-19 have dramatically impacted CSC services. Changes in staffing due to redeployment to medical units, childcare challenges, illness, and agency requirements to provide telehealth services from on-site locations have disrupted team functioning and clinical care. The rapid shift to telehealth without the benefit of planning, technology infrastructure, or training changed service delivery overnight. Telehealth may be challenging to implement in CSC due to common challenges young people with psychosis face including structural barriers (e.g., lack of equipment, WIFI, lack of privacy), cognitive deficits, symptoms of psychosis, and elevated suicide risk. However, staff innovation and creativity could transform these challenges into improvements and advantages. Teams have had to quickly adapt to online education, employment, and community systems to help participants adjust to changes required due to COVID-19 like physical distancing and interruption to school and work. Clinicians have had to develop strategies to assess and manage suicide risk via telehealth.

In 2019, OnTrackNY was awarded a hub within the Early Psychosis Intervention Network (EPINET) to advance a learning health care system (LHS) through enhanced stakeholder participation and improved data infrastructure. (7) The breadth of OnTrackNY sites coupled with OnTrack Central oversight provides a unique opportunity to examine the impacts of the COVID-19 crisis in New York State. The diversity of the 23 OnTrackNY teams located throughout the state enables examination of settings with a high and low prevalence of COVID-19 infections, and diverse regulatory and workforce environments. The OnTrackNY network includes programs that operate within variable settings and communities (outpatient clinics at community agencies, state-operated facilities, and community and academic hospitals in urban, suburban, and rural areas) with very diverse participant populations.

This project will examine the implications of modifications to service delivery within the OnTrackNY LHS during and after the COVID-19 crisis. We will use the implementation science framework, Framework for Reporting Adaptations and Modifications-Enhanced (FRAME), to systematically evaluate modifications and ascertain their impact. The FRAME captures processes for modification, including reasons for the modification, what was modified, level of modification, and timing across phases of treatment. (8) The FRAME’s detailed coding system will elucidate if modifications are fidelity-consistent with the CSC model and enhance service delivery, and whether any changes are associated with reduced effectiveness. (8)

## Methods/design

### Conceptual Framework

The FRAME uses a detailed coding system to capture modifications, including reasons for the modification, what was modified, level of modification, and timing across phases of treatment. (8) Our focus in accord with implementation and dissemination principles (9, 10) will be on multiple methods, multiple contextual levels and stakeholders, and pragmatic assessment strategies. (11) The FRAME will guide our mixed-method assessment to capture modifications to the delivery of CSC services and whether these are fidelity-consistent. (8) The perspectives of stakeholders on the acceptability, feasibility and perceived impact of these CSC modification will also be examined.

### Aims

This project aims to assess:

1. Implications of governmental and agency-level policy changes and decision-making that shifted the delivery of behavioral health services, and how these decisions impact OnTrackNY team staffing and functioning.
2. Implications for delivery of CSC services, including assessment of needed modifications by service component within the CSC model (e.g., medication management, psychotherapy, peer services, and supported employment and education services) and the phase of treatment from outreach/enrollment to discharge.
3. Impact on participant-level care processes (e.g., utilization of services) and outcomes (e.g., symptoms, social functioning, work/school participation, use of telehealth services, satisfaction with services) overall and within participant subgroups defined by COVID-19 impact (e.g., program modifications/adaptations, high or low intensity COVID-19 penetration) and other participant risk factors and characteristics (e.g., racial/ethnic disparities, suicide risk).

### Study design

We will utilize integrative mixed methods. (12) Qualitative interviews with multi-level stakeholders (program participants, families, providers, team leaders, agency leaders, and decision-makers at the state and local levels) will help us learn about the process that various stakeholders used to determine policy changes, modifications to CSC services, and their impact. Analysis of OnTrackNY program data will facilitate examining trends in team staffing and functioning, participant service utilization and care outcomes. We will accomplish study aims over three time periods, as indicated below (Table 1). Table 2 provides examples of areas to be studied and influenced by input from the integrative mixed-methods from Aims 1 and 2.

### Setting

The breadth of OnTrackNY sites provides an opportunity to examine the impacts of the COVID-19 crisis in New York State, which to date over 6% of the nation’s COVID-19 cases and 9% of deaths. (13) The diversity of the 23 OnTrackNY teams enables examination of changes in settings with a high and low prevalence of COVID-19 infections and diverse regulatory and workforce environments. The OnTrackNY network includes teams that operate within variable regulations (*e.g.*, outpatient clinics at ten community agencies, four state-operated facilities, and nine community and academic hospitals in urban, suburban, and rural areas).

### Characteristics of participants

Multi-level stakeholders, including program participants, families, providers, team leaders, agency leaders, trainers (OnTrack Central), and decision-makers at the state and local levels, will be interviewed. With the EPINET infrastructure for multi-stakeholder engagement, participants are recruited from three councils that represent OnTrackNY stakeholders, OnTrack Central and an EPINET executive committee that was convened to provide guidance and input on the OnTrackNY LHS.

#### Participants:

OnTrackNY serves young people between the ages of 16 and 30 who have experienced symptoms of non-affective psychosis for less than two years. Across the 23 teams, over 2200 people have received OnTrackNY treatment since the program began in 2013. Typically, young people are diagnosed with schizophrenia spectrum disorder. The average age of participants served is 21 years old, and 81% reside with family at admission to the program. The population served is diverse: 34% are non-Hispanic black, 26% Hispanic, and 25% non-Hispanic white. Furthermore, approximately 54% of participants have Medicaid, 35% have commercial insurance, and 4% are uninsured. The OnTrackNY participants’ diversity allows us to examine health equity within the context of a pandemic at multiple levels.

#### Youth and Young Adult Leadership Council (YYLC):

The YYLC (10–12 members) centers and elevates the voices and perspectives of youth and young adults who are either participants or graduates of OnTrackNY programs across New York State. Council members convene to represent and express what matters most to them in the design, delivery, and evaluation of OnTrackNY services and support youth-guided practices and principles in overall mental health systems transformation.

#### Family Advisory Council:

The Family Advisory Council (8–10 members) offers an opportunity for family members of OnTrackNY participants to give feedback on program services and materials and share ideas on how to increase family involvement in OnTrackNY programs.

#### Provider Council:

The Provider Council (6–8 members) is comprised of one provider from each of the seven role-based services. The goal of the council is to provide a forum for providers to ask questions about the OnTrackNY program, share their input on the improvement of care processes in the OnTrackNY program and EPINET data infrastructure initiatives and feedback on tools and resources for stakeholders.

#### OnTrack Central:

OnTrack Central (12 trainers) is an intermediary organization that provides training and technical assistance to the OnTrackNY sites.

#### EPINET executive committee:

This committee (8 members) includes state and local mental health officials, providers, payers, and youth and family advocates, and provides input and advice on issues affecting OnTrackNY teams and participants.

## Data collection

### Aim 1 (Exploration)

We will leverage the OnTrackNY stakeholder infrastructure to conduct semi-structured qualitative interviews with state/local decision-makers from New York State-Office of Mental Health, agency leadership, OnTrack Central trainers, and OnTrackNY team leaders. Using the FRAME, we will develop semi-structured interview guides for each stakeholder group to capture system-level decision-making that prompted service delivery modifications (e.g., shift to telehealth services). Interviewees will identify and briefly describe all modifications to behavioral health services during New York State’s stay at home order. (14) Interviewees will then identify the three most important modifications and their details, reasons for the modifications, and their importance. We will ask team leaders, agency leadership, and OnTrack Central trainers about their impact (e.g., positive/negative/no impact) on program staffing and functioning (e.g., location of staff, staff morale, supervision, and team coordination). The diversity of OnTrackNY sites (e.g., location, type of program, participant demographics) will allow exploration of larger contextual factors in administrative and organizational decision making across settings and how this affected team staffing and functioning.

Given the dynamic nature of adaptation, we will include ongoing, real-time tracking of modifications throughout all phases. Using the FRAME, we have a real-time tracking sheet to capture information learned from OnTrack Central role-based technical assistance calls with providers. A trained research assistant will review call notes and meet with OnTrack Central leadership weekly to track changes. These data will provide information directly from the front line of care. They will help identify new modifications, changes to previous modifications and see if they confirm, expand or elaborate upon what we are learning. This is a novel application of the FRAME that will capture a dynamic process in real-time.

### Aim 2 (Confirmation/Elaboration)

The OnTrackNY team leaders’ interviews will include questions about modifications by service component within the CSC model by phase of treatment. The FRAME will guide the development of semi-structured interview guides. Interviewees will identify modifications to the service components most relevant to their role. Then, they will identify the top three most important modifications and their details, reasons for the modifications and their importance. Detailed follow-up questions will assess acceptability (*e.g.*, comfort with telehealth sessions), feasibility (*e.g.*, access to computer or laptop for telehealth sessions), and perceived impact (*e.g.*, positive/negative or no impact) on care delivery.

We will conduct two focus groups with the OnTrackNY Family Advisory Council and with the Youth and Young Adult Leadership Council. Councils meet remotely to foster statewide participation. Using the FRAME, we will develop a focus group protocol to capture modifications to CSC service components. We will discuss the concept of modification with participants and ask about changes they experienced, for which service components, and why they think each change was made.

Based on our findings from the exploration phase, we will develop a set of questions about these major modifications (*e.g.*, telehealth) and their use, acceptability, feasibility, and perceived impact. These questions will be added to existing participant self-report every six months and to quarterly participant- and program-level data forms. We will also develop a family survey about the major modifications and share with families via the OnTrack Family Advisory Council and teams. We will validate and elaborate on survey findings via four remote focus groups with OnTrack Central leadership, the YYLC, the Family Advisory Council and Provider Council. Participants will be asked whether modifications are consistent with their care experience and how these may have evolved.

#### Fidelity review:

Modifications/adaptations from interviews and focus groups will be reviewed by the existing 4-member OnTrackNY Fidelity Committee which meets as needed to discuss team-level fidelity reports and plans for discussing fidelity assessments with teams. The OnTrackNY Fidelity tool and FRAME criteria for rating fidelity will be used to determine if a modification is fidelity-consistent (*i.e.*, whether the modification adheres to core CSC service components specified in the OnTrackNY Fidelity tool) or fidelity-inconsistent.

### Aim 3 (Examination)

OnTrackNY’s ongoing data collection infrastructure (program and participant-level) will be used to identify how (or if) the COVID-19 crisis has impacted changes in the population being served, how or which services are being offered and utilized, and participant outcome trajectories (Table 2). Analyses will further examine how changes in each of these areas are related to one another, using the natural experiment of the COVID-19 crisis to build evidence for how participant and service characteristics within the CSC model affect participant outcomes. While we will establish trends in program and participant characteristics across the pre-COVID period (since program inception in October 2013 through March 2020), we must remain flexible in our analysis of the post March 2020 period to account for additional key dates (e.g., dates when stay at home order ends in given regions, or the vaccine is distributed).

## Statistical methods

### Aim 1

Interviews and focus groups will be audio-recorded, transcribed and coded by two independent coders. Qualitative content will be managed using Atlas.ti software. Directed content analysis is a deductive analytical approach that derives codes from existing theories, frameworks and constructs and is used to validate, expand, or refute theories. (15, 16) We will use FRAME dimensions to develop our initial codes and list of major modifications identified by stakeholders commenting on 1) system-level decision making and its impact on team staffing and functioning; and 2) modifications to CSC service components. The list of major modifications from interviews and focus groups will be reviewed alongside a list of modifications generated by the ongoing, real-time tracking of modifications. In the case of divergence in type and characteristics of existing modifications or emerging modifications, the coders and study team will reconcile and revise the major modifications list. Questions to confirm major modifications and their use, acceptability, feasibility, and impact will be added to existing participant self-report, and other participant- and program-level data collection. A new survey to obtain the family perspective will be developed as part of Aim 2.

Exploring variability in modifications across the OnTrackNY network: To understand how system-level decisions impacted OnTrackNY team functioning and differences across the network, we will examine our qualitative findings using a thematic matrix analytical approach. (17, 18) This analytical process will help organize findings from each of our qualitative data sources into a matrix to compare side-by-side the different types of modification identified by stakeholders and their perceived impact. We will then generate a list of relevant system, organizational, staff, and participant-level modifications by the level of consensus (*i.e.*, identified by more than one source) and operational salience (*i.e.*, identified as critical). Using the same thematic matrix analytical approach, we will generate a list of relevant modifications based on OnTrackNY dimensions (location, type of program, and characteristics of participant population served) by the level of consensus (*i.e.*, identified by more than one source) and operational salience (*i.e.*, identified as critical).

### Aim 2

Descriptive analyses of surveys will be conducted by stakeholder group and will explore differences in use, acceptability, feasibility and perceived impact by different OnTrackNY dimensions (location, type of program, respondent characteristics including demographics, age, time in the program). The list of major modifications from the surveys will be reviewed alongside a list of modifications generated by the ongoing, real-time tracking of modifications. In the case of divergence in type and characteristics of existing modifications or emerging modifications, the coders and study team will reconcile and revise the list of the major modifications. Modifications that meet threshold criteria (at least 50% of all stakeholder groups identified) will be considered tier 1 modifications and used in subsequent quantitative analyses examining effects on participant-level care processes and outcomes in Aim 3 work. Modifications that do not meet these threshold criteria (tier 2 modifications) will be summarized and described based on OnTrackNY dimensions by level of operational salience.

### Aim 3

As in prior work with OnTrackNY longitudinal data,(4, 19, 20) we will utilize generalized linear mixed models which account for the natural multi-level structure of the data of repeated measures on participants (every 90 days by providers and 6-months participant self-report) nested within teams. Importantly, we will incorporate a fixed time period indicator of the pre-March 2020 period allowing for a categorical indicator for each calendar month post-March 2020 until the end of the study period (likely first quarter of 2022). Outcomes and predictors will vary depending on the question (Table 2). Still, in all cases, we will use best statistical modeling practices for estimating and testing effects (focusing on effect sizes and uncertainty using 95% confidence intervals). *Statistical power:* With over 2200 participants served to date, 23 different teams across the state, over 500 new enrollees in 2019, and the program continually growing, we have adequate power to address questions of change. For example, for questions regarding the population served, we have 80% power to detect a shift in early discharge of participants (*i.e.*, disengagement before one year, historically at ~ 25%), (21) as small as 7%. Power is similar for detecting changes in participant outcomes, e.g., percent of participants in work or education consistently for the whole year in the pre period as compared to post March 2020. For assessing associations between team level characteristics (*e.g.*, modifications) and frequency of participants receiving services, we have 80% power to detect at least medium effect sizes (Cohen’s h = 0.40) assuming intraclass correlation (ICC = 0.10) historically found for OTNY outcomes within teams. Finally, the diversity of the participants and teams across the state provide adequate power to examine racial minority subgroups and geographic areas with high and low COVID penetration (New York City vs. upstate areas).

## Development of the CSC Model Adaptation Guide

Lessons learned will be reviewed with the EPINET executive committee, and the OnTrackNY YYLC, Family Advisory, and Provider Councils and study findings will be summarized in a *CSC Model Adaptation Guide*, with findings that are consistent with fidelity and favorable outcomes of care. This guide will inform CSC programs, as well as the state and local mental health authorities to which they are accountable, regarding modification and impact on care process and participant outcomes. It will thus be useful on a clinical and policy level. It will also inform the development of tailored technical assistance that CSC programs need within OnTrackNY, the EPINET network, and CSC programs nationally

## Discussion

The rapid rise of COVID-19 created shocks to the health care system. To combat the rapid rise of infections, the New York State (NYS) stay at home order (effective March 22, 2020) required all non-essential businesses to cease in-person operations, required people to shelter in place, and move to distance technology. (14) Engagement with the OnTrackNY network revealed how these conditions have dramatically impacted CSC services. For example, many sites rapidly moved from providing 0% to nearly 100% telehealth with no time to plan, and with limited infrastructure, experience, or training. Agencies faced redeployment of staff to medical units, staff members’ childcare challenges, illness of team members as well as agency requirements regarding the need to be on-site, which caused staff changes and disruptions. Some participants faced access barriers (limited availability of cell phones, WIFI). Moreover, telehealth may be particularly challenging in CSC treatment, with participants experiencing cognitive deficits and symptoms of psychosis, (22) as well as suicide risk. Despite these challenges, OnTrack Central has begun to learn about staff innovation and creativity that could transform these challenges to improvements and advantages. OnTrackNY teams remain committed to their mission to provide high-quality care to improve participants’ outcomes.

This crisis provides an opportunity to examine how CSC teams, facing a substantial and unexpected disruption, can respond and adapt to the changing needs of participants, families, and their own agencies, to maintain high-quality services. The health care and economic effects of the COVID-19 crisis are likely to persist for some time. Some changes in health care delivery, particularly increased use of telehealth may be permanent. It is crucial to understand the impact of these changes on service delivery and on participant outcomes. (23) This is a unique opportunity to understand how decisions to adapt service delivery are made, how they are implemented in response to the crisis and how they impact different stakeholder groups, including policy leaders, providers, participants and family members. This study provides a unique opportunity to examining this system transformation by systematically capturing service modifications using implementation science tools and dynamically examining this process as it unfolds.

Our study is limited by the restricted geographic area of New York State, although the sample includes individuals from a wide range of geographic areas and economic resources. It may have an over-representation of individuals with early psychosis at the highest need and those who are or have received services as part of the OnTrackNY program. Further, we will be obtaining information from people retrospectively regarding their past experiences, which will be subject to recall bias, even as the landscape changes moving forward.

We expect that our study will generate questions for future research within the learning healthcare system framework. For example, we expect that the COVID-19 pandemic is likely to exacerbate known racial and ethnic disparities in care and outcomes; this work will help to generate hypotheses for strategies to reduce inequities (24) such as access to remote behavioral healthcare. We anticipate that we will learn about addressing suicidality and other high-risk clinical situations. Finally, we expect that lessons learned from this study will inform behavioral health services and their implementation (25) more broadly with implications for other community-oriented team-based models such as Assertive Community Treatment, supported employment, and peer services. The breadth and depth of OnTrackNY combined with the infrastructure and expertise supported by EPINET provides a foundation for a unique, timely and high-impact study.

## Supplementary Material

Supplement 1

Supplement 2

Supplement 3

## Figures and Tables

**Table 1:** Study Aims

Study Aims	Purpose	Methods	Stakeholder Groups Included
Aims 1 &2	Exploration	Interviews	OnTrackNY providers and team leaders; Agency leadership; OnTrack Central trainers; State and local-level decision makers
		Focus groups	OnTrackNY Youth, Family Advisory and Provider Councils
Aim 2	Confirmation Elaboration	Data collection Survey Fidelity review Focus groups	Participant self-report; Participant-level data collection (OnTrackNY primary clinician); Program-level data collection (OnTrackNY team leaders) Family survey OnTrackNY fidelity committee OnTrack Central leadership, OnTrackNY Youth and Young Adult Leadership, Family Advisory and Provider Councils
Aims 1–3	Exploration, Confirmation, Elaboration	Tracking Sheet	OnTrack Central technical assistance calls
Aims 2 &3	Examination	Data analysis	OnTrackNY clinicians and participants

**Table 2:** Data analyses examining the impact on care processes and outcomes (pre versus post-March 2020)

Example areas to be studied	Outcomes monitored	Key features and subgroups associated with outcomes	Data Sources
Any changes in the population served	Program level rates of enrollment and discharge across OTNY	Geographic areas with high and low COVID penetration.	Clinician-reported data aggregated to the program level
	Participant level characteristics at enrollment to OTNY	Referral source (e.g., inpatient unit), duration of untreated psychosis (DUP), symptoms, additional variables as identified in Aims 1 and 2	Clinician-reported data and participant self-report data
	Participant features at time of discharge/disengagement from OTNY	Length of stay in OnTrackNY; symptoms, occupational and social functioning; use of telehealth services; satisfaction with services; other factors as identified in Aims 1 and 2	Clinician-reported data and participant self-report data
Any changes in how and which services are implemented	Frequency of utilization of OTNY services overall and with particular team members (e.g., peer support, SEES)	Presence of identified modifications in a clinic (e.g., telehealth interventions), programs where staffing was affected (e.g., FTE per team), participant utilization of modifications, fidelity to the CSC model, other factors identified in Aims 1 and 2	Program-level data and clinician-reported data
Any changes in participant outcomes	Symptoms, social and occupational functioning, work/school participation, suicidality, and other factors identified in Aims 1 and 2	Clinical characteristics at enrollment, age, gender, race/ethnicity, DUP, educational attainment, program site, presence of identified modifications, other factors as identified in Aims 1 and 2	Clinician and participant self-reported data

## List Of Abbreviations

CSC: Coordinated Specialty Care
EPINET: Early Psychosis Intervention Network
LHS: Learning health care system
FRAME: Framework for Reporting Adaptations and Modifications-Enhanced
CBTp: Cognitive Behavioral Therapy for psychosis
NYS: New York State
YYLC: Youth and Young Adult Leadership Council
DUP: Duration of untreated psychosis
FTE: Full time equivalent
