# Supplementary material for: Adapting Coordinated Specialty Care in the Post-COVID-19 Era: Study Protocol for an Integrative Mixed-methods Study
Source: Res Sq. 2021 Apr 26:rs.3.rs-452200. Preprint. [Version 1] doi: 10.21203/rs.3.rs-452200/v1 (PMC8132251; doi:10.21203/rs.3.rs-452200/v1)
Supplement: Supplement 2 [file a4050d67e08753a193212910.pdf]

NEW YORK STATE PSYCHIATRIC INSTITUTE
INSTITUTIONAL REVIEW BOARD
MEMORANDUM

July 17, 2019

To: Dr. Lisa Dixon

From: Corinne Rogers, MS, CIP
Director, IRB

Subject: Project # **7840: OnTrackNY's Learning Healthcare System**

The IRB office has reviewed the information you provided regarding the above project. Your role and the current activities (Program evaluation/quality improvement), do not meet the definition of human subjects research requiring IRB review according to the federal definition 45CFR46.102 (f).

A determination of "Not Human Subjects Research" does not absolve individuals conducting the activity of any ethical and legal responsibilities and obligations that may apply. Be advised precautions should still be taken to maintain requirements that have been set forth by institutional security policies and HIPAA regulations.

If the scope of the work described changes, you are required to inform the IRB and provide additional information for review before the modified project can be initiated. At such time as definite plans for the involvement of human subjects are developed, a new application will need to be submitted in PRISM to the IRB for review and approval prior to the involvement of human participants.

Please retain a copy of this letter for your files as reference. You may contact the IRB with any questions at 646-774-7155 or by email at IRBMail@nyspi.columbia.edu. Thank you.

Signed copy in IRB file
