# Supplementary material for: Adapting Coordinated Specialty Care in the Post-COVID-19 Era: Study Protocol for an Integrative Mixed-methods Study
Source: Res Sq. 2021 Apr 26:rs.3.rs-452200. Preprint. [Version 1] doi: 10.21203/rs.3.rs-452200/v1 (PMC8132251; doi:10.21203/rs.3.rs-452200/v1)
Supplement: Supplement 3 [file caca0dd0b088b4a7c3d3c3df.pdf]

RESEARCH **Federal Award Date:** 08/20/2020
Department of Health and Human Services
National Institutes of Health

NATIONAL INSTITUTE OF MENTAL HEALTH

Grant Number: 3R01MH120597-02S1
FAIN: R01MH120597

Principal Investigator(s):
LISA B. DIXON (contact), MD
Jennifer Humensky, PHD

Project Title: Adapting Coordinated Specialty Care in the Post COVID-19 Era

Greenhill, Janelle Rene
Director of Administration
NYPI 1051 Riverside Drive
New York, NY 100321007

Award e-mailed to: nga@nyspi.columbia.edu

Period Of Performance:
Budget Period: 08/18/2020 – 07/31/2021
Project Period: 08/15/2019 – 07/31/2024

Dear Business Official:

The National Institutes of Health hereby awards a grant in the amount of $324,329 (see “Award Calculation” in Section I and “Terms and Conditions” in Section III) to Research Foundation for Mental Hygiene in support of the above referenced project. This award is pursuant to the authority of 42 USC 241 42 CFR 52 and is subject to the requirements of this statute and regulation and of other referenced, incorporated or attached terms and conditions.

Acceptance of this award including the “Terms and Conditions” is acknowledged by the grantee when funds are drawn down or otherwise obtained from the grant payment system.

Each publication, press release, or other document about research supported by an NIH award must include an acknowledgment of NIH award support and a disclaimer such as “Research reported in this publication was supported by the National Institute Of Mental Health of the National Institutes of Health under Award Number R01MH120597. The content is solely the responsibility of the authors and does not necessarily represent the official views of the National Institutes of Health.” Prior to issuing a press release concerning the outcome of this research, please notify the NIH awarding IC in advance to allow for coordination.

Award recipients must promote objectivity in research by establishing standards that provide a reasonable expectation that the design, conduct and reporting of research funded under NIH awards will be free from bias resulting from an Investigator’s Financial Conflict of Interest (FCOI), in accordance with the 2011 revised regulation at 42 CFR Part 50 Subpart F. The Institution shall submit all FCOI reports to the NIH through the eRA Commons FCOI Module. The regulation does not apply to Phase I Small Business Innovative Research (SBIR) and Small Business Technology Transfer (STTR) awards. Consult the NIH website http://grants.nih.gov/grants/policy/coi/ for a link to the regulation and additional important information.

If you have any questions about this award, please contact the individual(s) referenced in Section IV.

Sincerely yours,

Tamara A. Kees
Grants Management Officer
NATIONAL INSTITUTE OF MENTAL HEALTH

Additional information follows

SECTION I – AWARD DATA – 3R01MH120597-02S1

Award Calculation (U.S. Dollars)

**Salaries and Wages**	$121,082
**Fringe Benefits**	$31,884
**Personnel Costs (Subtotal)**	$152,966
**Consultant Services**	$7,200
**Materials & Supplies**	$1,500
**Other**	$10,581
**Subawards/Consortium/Contractual Costs**	$31,426
**Federal Direct Costs**	$203,673
**Federal F&A Costs**	$120,656
**Approved Budget**	$324,329
**Total Amount of Federal Funds Obligated (Federal Share)**	$324,329
**TOTAL FEDERAL AWARD AMOUNT**	$324,329
**AMOUNT OF THIS ACTION (FEDERAL SHARE)**	$324,329

SUMMARY TOTAL FEDERAL AWARD AMOUNT YEAR (2)	
**GRANT NUMBER**	**TOTAL FEDERAL AWARD AMOUNT**
3R01MH120597-02S1	$324,329
5R01MH120597-02	$1,587,234
**TOTAL**	**$1,911,563**

SUMMARY TOTALS FOR ALL YEARS		
**YR**	**THIS AWARD**	**CUMULATIVE TOTALS**
2	$324,329	$1,911,563
3	$305,233	$1,874,619
4	$0	$1,562,663
5	$0	$1,554,777

Recommended future year total cost support, subject to the availability of funds and satisfactory progress of the project

Fiscal Information:

CFDA Name: Mental Health Research Grants
CFDA Number: 93.242
EIN: 1141410842A2
Document Number: RMH120597A
PMS Account Type: P (Subaccount)
Fiscal Year: 2020

IC	CAN	2020	2021
OD	8045531	$324,329	
MH	8472591		$305,233

Recommended future year total cost support, subject to the availability of funds and satisfactory progress of the project

NIH Administrative Data:
PCC: 85-TPAS1 / **OC**: 41023 / **Released**: KEEST 08/19/2020
Award Processed: 08/20/2020 12:18:15 AM

SECTION II – PAYMENT/HOTLINE INFORMATION – 3R01MH120597-02S1

For payment and HHS Office of Inspector General Hotline information, see the NIH Home Page at http://grants.nih.gov/grants/policy/awardconditions.htm

SECTION III – TERMS AND CONDITIONS – 3R01MH120597-02S1

This award is based on the application submitted to, and as approved by, NIH on the above-titled project and is subject to the terms and conditions incorporated either directly or by reference in the following:

a. The grant program legislation and program regulation cited in this Notice of Award.
b. Conditions on activities and expenditure of funds in other statutory requirements, such as those included in appropriations acts.
c. 45 CFR Part 75.
d. National Policy Requirements and all other requirements described in the NIH Grants Policy Statement, including addenda in effect as of the beginning date of the budget period.
e. Federal Award Performance Goals: As required by the periodic report in the RPPR or in the final progress report when applicable.
f. This award notice, INCLUDING THE TERMS AND CONDITIONS CITED BELOW.

(See NIH Home Page at http://grants.nih.gov/grants/policy/awardconditions.htm for certain references cited above.)

Research and Development (R&D): All awards issued by the National Institutes of Health (NIH) meet the definition of "Research and Development" at 45 CFR Part§ 75.2**.** As such, auditees should identify NIH awards as part of the R&D cluster on the Schedule of Expenditures of Federal Awards (SEFA). The auditor should test NIH awards for compliance as instructed in Part V, Clusters of Programs. NIH recognizes that some awards may have another classification for purposes of indirect costs. The auditor is not required to report the disconnect (i.e., the award is classified as R&D for Federal Audit Requirement purposes but non-research for indirect cost rate purposes), unless the auditee is charging indirect costs at a rate other than the rate(s) specified in the award document(s).

An unobligated balance may be carried over into the next budget period without Grants Management Officer prior approval.

This grant is subject to Streamlined Noncompeting Award Procedures (SNAP).

This award is subject to the requirements of 2 CFR Part 25 for institutions to receive a Dun & Bradstreet Universal Numbering System (DUNS) number and maintain an active registration in the System for Award Management (SAM). Should a consortium/subaward be issued under this award, a DUNS requirement must be included. See http://grants.nih.gov/grants/policy/awardconditions.htm for the full NIH award term implementing this requirement and other additional information.

This award has been assigned the Federal Award Identification Number (FAIN) R01MH120597. Recipients must document the assigned FAIN on each consortium/subaward issued under this award.

Based on the project period start date of this project, this award is likely subject to the Transparency Act subaward and executive compensation reporting requirement of 2 CFR Part 170. There are conditions that may exclude this award; see http://grants.nih.gov/grants/policy/awardconditions.htm for additional award applicability information.

In accordance with P.L. 110-161, compliance with the NIH Public Access Policy is now mandatory. For more information, see NOT-OD-08-033 and the Public Access website: http://publicaccess.nih.gov/.

This award provides support for one or more clinical trials. By law (Title VIII, Section 801 of Public Law 110-85), the "responsible party" must register "applicable clinical trials" on the ClinicalTrials.gov Protocol Registration System Information Website. NIH encourages registration of all trials whether required under the law or not. For more information, see http://grants.nih.gov/ClinicalTrials_fdaaa/

This award is funded by the following list of institutes. Any papers published under the auspices of this award must cite the funding support of all institutes.

Office Of The Director, National Institutes Of Health (OD)

In accordance with the regulatory requirements provided at 45 CFR 75.113 and Appendix XII to 45 CFR Part 75, recipients that have currently active Federal grants, cooperative agreements, and procurement contracts with cumulative total value greater than $10,000,000 must report and maintain information in the System for Award Management (SAM) about civil, criminal, and administrative proceedings in connection with the award or performance of a Federal award that reached final disposition within the most recent five-year period. The recipient must also make semiannual disclosures regarding such proceedings. Proceedings information will be made publicly available in the designated integrity and performance system (currently the Federal Awardee Performance and Integrity Information System (FAPIIS)). Full reporting requirements and procedures are found in Appendix XII to 45 CFR Part 75. This term does not apply to NIH fellowships.

Treatment of Program Income:
Additional Costs

SECTION IV – MH Special Terms and Conditions – 3R01MH120597-02S1

Clinical Trial Indicator: Yes
This award supports one or more NIH-defined Clinical Trials. See the NIH Grants Policy Statement Section 1.2 for NIH definition of Clinical Trial.

RESTRICTION: ADMINISTRATIVE SUPPLEMENT:
This award provides an administrative supplement in the amount of $324,329 total costs ($203,673 direct costs + $120,656 facilities and administrative costs) to examine the impacts of the COVID-19 crisis in New York State, per recipient's request dated 06//15/2020. These funds may not be used for any other purpose without the prior written approval of NIMH staff. Funds carried over will remain restricted for the stated purpose only. Any ultimate balance of the restricted funds must be specifically identified on the Federal Financial Report (if applicable).

Future year support for this supplement is reflected below:

YEAR	AMOUNT	BUDGET PERIOD
-03	$305,233	08/01/2021 - 07/31/2022

AWARD NOTICE:
This award has been made in response to the application submitted under the Funding Opportunity Announcement NOT-MH-20-047, which can be referenced at: https://grants.nih.gov/grants/guide/notice-files/NOT-MH-20-047.html.

DATA AND SAFETY MONITORING:
The level and frequency of human subject data and safety monitoring should always be commensurate with the risk and nature of the research. Human subject data and safety monitoring for this grant is the responsibility of the Principal Investigator(s) and the IRB of record for this grant. The recipient is reminded of required reporting pursuant to NIHGPS 4.1.15.3 and the NIMH Reportable Events Policy cited in NOT-MH-19-027, as appropriate.

CONSORTIUM / CONTRACTUAL COSTS:
This award includes funds for consortium activity with **Columbia University and Washington University St. Louis**. Each consortium is to be established and administered in accordance with the NIH Grants Policy Statement (http://grants.nih.gov/grants/policy/nihgps/index.htm). No foreign performance site may be added to this project without the written prior approval of the National Institute of Mental Health.

STAFF CONTACTS

The Grants Management Specialist is responsible for the negotiation, award and administration of this project and for interpretation of Grants Administration policies and provisions. The Program Official is responsible for the scientific, programmatic and technical aspects of this project. These individuals work together in overall project administration. Prior approval requests (signed by an Authorized Organizational Representative) should be submitted in writing to the Grants Management Specialist. Requests may be made via e-mail.

Grants Management Specialist: Betty Nkansah
Email: bnkansah@mail.nih.gov **Phone**: 301-443-7638 **Fax**: 301-480-1956

Program Official: Matthew V. Rudorfer
Email: mrudorfe@mail.nih.gov **Phone**: 301-443-1111 **Fax**: 301-443-4045

SPREADSHEET SUMMARY
GRANT NUMBER: 3R01MH120597-02S1

INSTITUTION: Research Foundation for Mental Hygiene

Budget	Year 2	Year 3	Year 4	Year 5
Salaries and Wages	$121,082	$121,082		
Fringe Benefits	$31,884	$31,884		
Personnel Costs (Subtotal)	$152,966	$152,966		
Consultant Services	$7,200	$7,200		
Materials & Supplies	$1,500			
Other	$10,581	$4,852		
Subawards/Consortium/Contractual Costs	$31,426	$31,427		
TOTAL FEDERAL DC	$203,673	$196,445		
TOTAL FEDERAL F&A	$120,656	$108,788		
TOTAL COST	$324,329	$305,233	$0	$0

Facilities and Administrative Costs	Year 2	Year 3	Year 4	Year 5
F&A Cost Rate 1	59.1%	59.1%		
F&A Cost Base 1	$196,168	$176,087		
F&A Costs 1	$115,935	$104,067		
F&A Cost Rate 2	62.9%	62.9%		
F&A Cost Base 2	$7,505	$7,505		
F&A Costs 2	$4,721	$4,721		
